# Supplementary material for: Active site specificity profiling datasets of matrix metalloproteinases (MMPs) 1, 2, 3, 7, 8, 9, 12, 13 and 14
Source: Data Brief. 2016 Feb 22;7:299–310. doi: 10.1016/j.dib.2016.02.036 (PMC4777984; doi:10.1016/j.dib.2016.02.036)
Supplement: Supplementary file 10 — Supplementary material [file mmc10.zip › WebPICS_hMMP2_G_1%/P1.html]

 

PICS results


|  |  |
| --- | --- |
| **P1\_A**  34 in 246 sites   13.8 %    effects > 10 perc. pnts.  (vice-versa in brackets)  P3\_A: 13.1 (13.5) |  |
| **P1\_D**  22 in 246 sites   8.9 %    effects > 10 perc. pnts.  (vice-versa in brackets)  P3prime\_N: 10.9 (13.3) |  |
| **P1\_G**  37 in 246 sites   15.0 %    effects > 10 perc. pnts.  (vice-versa in brackets)  P3\_I: 10.9 (12.3)   P2prime\_N: 17.5 (65.0)   P3prime\_V: 18.7 (25.7) |  |
| **P1\_H**  7 in 246 sites   2.8 %    effects > 10 perc. pnts.  (vice-versa in brackets)  P3prime\_K: 35.6 (13.9) |  |
| **P1\_N**  23 in 246 sites   9.3 %    effects > 10 perc. pnts.  (vice-versa in brackets)  P3\_I: 17.0 (11.9)   P3\_L: 15.5 (13.8)   P2prime\_F: 11.3 (17.4)   P2prime\_Q: 11.7 (19.3) |  |
| **P1\_Q**  11 in 246 sites   4.5 %    effects > 10 perc. pnts.  (vice-versa in brackets)  P2prime\_M: 15.4 (24.1) |  |
| **P1\_S**  30 in 246 sites   12.2 %    effects > 10 perc. pnts.  (vice-versa in brackets)  P2prime\_I: 19.1 (16.4) |  |
